# Efficient Extraction of Phenolic Compounds From *Cariniana rubra* Gardener Ex Miers From the Legal Amazon: Ultrasound‐Assisted Optimization, Chemical Characterization, and Antioxidant Potential

**DOI:** 10.1002/cbdv.202403256

**Published:** 2025-07-17

**Authors:** Aline Biggi Maciel, Elis Ramos de Queiroz Jácome, Naísa Andrade da Silva, Rachel de Moura Nunes Fernandes, Claudia Andrea Lima Cardoso, Elisandra Scapin

**Affiliations:** ^1^ Graduate Program in Biodiversity and Biotechnology—BIONORTE Federal University of Tocantins, Palmas Campus Palmas Tocantins Brazil; ^2^ Laboratory of Teaching and Research in Natural Products and Biomass Federal University of Tocantins Palmas Tocantins Brazil; ^3^ Chemistry Laboratory Federal University of Tocantins Palmas Tocantins Brazil; ^4^ Center For Studies in Natural Resources State University of Mato Grosso Do Sul Dourados Mato Grosso do Sul Brazil

**Keywords:** antioxidants, jequitibá‐vermelho, phytochemistry, response surface methodology, total phenolic compounds

## Abstract

*Cariniana rubra*, native to the Brazilian Cerrado and the Amazon‐Cerrado ecotone, is traditionally used in folk medicine but remains underexplored. This study optimized extraction yield and bioactive compound content using ultrasound‐assisted extraction (UAE) and Soxhlet extraction. A central composite rotational design (CCRD) assessed ethanol concentration and extraction time, with response surface modeling (RSM) determining optimal conditions. UAE yielded 49.60% (50% ethanol, 132 min) and Soxhlet 25.25% (78% ethanol, 300 min). Maximum phenolic compound contents were 382.62 mg GAE g^−1^ (UAE; 48 min) and 385.95 mg GAE g^−1^ (Soxhlet; 480 min). Flavonoid contents were 137.83 mg QE g^−1^ (UAE; 90 min) and 73.66 mg QE g^−1^ (Soxhlet; 120 min). Antioxidant activity was confirmed by DPPH•, with IC_50_ values of 37.73 µg mL^−1^ (UAE) and 36.20 µg mL^−1^ (Soxhlet), surpassing Trolox (23.64 µg mL^−1^), and equivalent to Trolox in ABTS assays (53.67 and 38.67 µM Trolox g^−1^) and FRAP (453.44 and 558.44 µM Fe^2+^ g^−1^). FT‐IR analysis confirmed phenolic compound indicators, while LC–DAD identified epicatechin, rutin, and myricetin. These optimized methods produced extracts with high antioxidant activity, suggesting potential for further applications.

## Graphical abstract

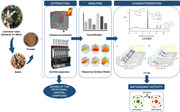

## 1 Introduction

Brazil is globally recognized for its vast plant biodiversity, distributed across six major biomes, including the Amazon, widely regarded as one of the most important ecosystems on Earth, and the Atlantic Forest and Cerrado, both identified as global biodiversity hotspots [1]. The transition zone between the Amazon and Cerrado, known as the ecotone, holds significant ecological importance due to its unique combination of species from both biomes. This region is rich in biological diversity, making it a critical area for conservation efforts [2].

One notable species within the Cerrado and the Amazon‐Cerrado ecotone is *Cariniana rubra* Gardner ex Miers (*C. rubra*), a member of the Lecythidaceae family, commonly known as *jequitibá*, *cachimbeira*, or *jequitibá‐vermelho*. Ethnopharmacological reports suggest that the bark of *C. rubra* has been traditionally used to treat throat infections, ovarian disorders, and venereal diseases [3]. It is also employed in the treatment of kidney ailments [4] and has demonstrated antibacterial, antifungal, antioxidant [5], antinociceptive, and antipyretic activities [6]. These bioactivities are largely attributed to the presence of phenolic compounds [7].

Phenolic compounds are a diverse class of organic molecules found throughout plant tissues, characterized by one or more hydroxyl groups (─OH) attached to an aromatic ring [8]. Their chemical structure underpins their vital roles in plant growth, development, reproduction, and defense [9, 10, 11]. The structural diversity of phenolics—including phenolic acids, flavonoids, coumarins, stilbenes, tannins, lignans, and lignins—enables a broad range of biological activities, with antioxidant properties being especially notable [10]. These activities are linked to their redox capabilities, allowing phenolics to act as reducing agents, hydrogen donors, and free radical scavengers [11].

Given the bioactive potential of plant compounds, bioprospecting plays a crucial role in identifying and quantifying these compounds, as well as in promoting the conservation of native plants and supporting sustainable regional development [12]. The effectiveness of bioactive compound extraction is highly dependent on the efficiency of the chosen extraction techniques, solvent types, and the specific plant parts used [13, 14].

Traditional Soxhlet extraction, despite its advantages, may cause thermal degradation of heat‐sensitive compounds due to the high extraction temperatures (50°C–90°C) and extended extraction times, often resulting in low yields [15]. In contrast, ultrasound‐assisted extraction (UAE) has emerged as a promising alternative, offering a more sustainable approach by reducing solvent consumption, simplifying handling, shortening extraction times, and enhancing yields at lower costs [16, 17].

The efficiency of bioactive compound extraction is influenced by several experimental factors. The application of mathematical models, such as response surface methodology (RSM), allows for the prediction of variable responses while exploring their interactions, thus identifying optimal extraction conditions [18]. In this study, optimizing UAE and Soxhlet extraction methods for *C. rubra* is key to maximizing yield and enhancing the concentration of bioactive compounds, promoting a more sustainable use of this species' bark. This not only facilitates the rational use of plant resources but also enhances the therapeutic potential of the extracts, which may be used as adjuvants in treating diseases traditionally associated with the species, further contributing to the conservation and valorization of native species.

The objective of this study is to determine the optimal conditions and the most effective method for extracting bioactive compounds from the bark of *C. rubra*. In addition, we aim to evaluate how extraction time and ethanol content influence the antioxidant capacity of the extracts and to perform a detailed chemical characterization, given the plant's ethnopharmacological significance and the limited scientific research available on this species.

## 2 Results and Discussion

### 2.1 Yield, Phenolic, and Flavonoid Content

The results obtained for the yield, total phenolic, and flavonoid content in the bark extracts of *C. rubra*, obtained through UAE (UBT1–UBT11) and Soxhlet extraction (SBT1–SBT11), are presented in Table 1.

**TABLE 1:** Values obtained for yield (*Y*, expressed as %), total phenolic content (PC, expressed in milligrams of extract per gram of gallic acid equivalent, mg GAE g^−1^), and flavonoid content (FC, expressed in milligrams of extract per gram of rutin equivalent, mg RE g^−1^) for *Cariniana rubra* bark extracts obtained by ultrasound‐assisted extraction (UAE) and Soxhlet apparatus.

Ultrassom assisted				Soxhlet			
Extract	*Y* (%)	PC (mg GAE g^−1^)	FC (mg RE g^−1^)	Extract	*Y* (%)	PC (mg GAE g^−1^)	FC (mg RE g^−1^)
UBT1	25.55	198.77 ± 2.7^gB^	36.72 ± 3.4^dC^	SBT1	5.90	325.18 ± 6.0^eA^	17.83 ± 3.3^fD^
UBT2	43.31	321.85 ± 4,7^dB^	49.50 ± 5.0^cC^	SBT2	16.42	372.87 ± 1.9^bA^	25.61 ± 4.6^eD^
UBT3	26.90	356.97 ± 5.8^bA^	99.22 ± 3.8^bC^	SBT3	7.10	240.05 ± 3.8^iB^	73.66 ± 1.7^aD^
UBT4	43.80	332.62 ± 2.3^cB^	31.17 ± 3.0^dC^	SBT4	19.97	385.95 ± 1.2^aA^	35.33 ± 3.3^dC^
UBT5	28.90	382.62 ± 4.7a^A^	26.72 ± 5.1^dD^	SBT5	14.35	251.08 ± 5.4^hB^	65.61 ± 5.4^bC^
UBT6	49.60	204.92 ± 4.8^fB^	40.61 ± 5.9^cD^	SBT6	19.75	355.44 ± 1.2^dA^	73.11 ± 3.8^aC^
UBT7	26.69	334.67 ± 1.9^cA^	19.22 ± 1.7^eD^	SBT7	9.95	246.46 ± 3.8^hB^	32.56 ± 0.5^dC^
UBT8	24.62	356.21 ± 7.3^bB^	137.83 ± 5.0a^C^	SBT8	25.25	367.23 ± 2.3^cA^	35.89 ± 3.8^dD^
UBT9	42.65	202.87 ± 5.8^fB^	29.22 ± 2.9^dD^	SBT9	13.33	311.59 ± 1.9^fA^	40.06 ± 2.1^cC^
UBT10	41.18	205.44 ± 5.5^fB^	32.00 ± 2.5^dD^	SBT10	14.33	300.56 ± 5.4^gA^	39.22 ± 1.3^cC^
UBT11	41.72	223.64 ± 5.1^eB^	35.61 ± 5.5^dC^	SBT11	14.75	321.85 ± 2.3^eA^	40.33 ± 2.5^cC^

*Note*: Values represent the mean ± SD (*n* = 3). Different lowercase letters above the lines and uppercase letters between the columns indicate significant differences (*p* < 0.05, ANOVA followed by Tukey's test).

Abbreviations: SD, standard deviation; SBT, Soxhlet bark testing; UBT, ultrasonic bark testing.

The total extract yields obtained via UAE ranged from 24.62% to 49.60% (total yield of the destruction process, calculation based on the initial dry weight of the raw material used and the final weight of the extraction obtained) demonstrating a significantly higher efficiency when compared to Soxhlet extraction, which yielded between 5.9% and 25.25%.

Dhanani et al. [19] highlighted that UAE is an effective alternative for improving extraction yields. In their study, they compared UAE (11.85%) with Soxhlet extraction (9.51%). The increased yield in UAE can be attributed to the combined effects of various mechanisms, particularly acoustic cavitation. This phenomenon induces processes such as fragmentation and localized erosion, which promote the rupture of the plant cell walls, increasing the interaction between the extracted material and the solvent. As a result, there is enhanced release of bioactive compounds, ultimately increasing the extraction yield [17, 20, 21].

For total phenolic content, the extracts from *C. rubra* bark obtained by UAE showed values ranging from 198.77 to 382.62 mg EAG g^−1^, while Soxhlet extracts had values between 240.05 and 385.95 mg EAG g^−1^. The sample UBT5, extracted by UAE for 48 min with 50% ethanol, exhibited the highest phenolic content (382.62 ± 4.7 mg EAG g^−1^) for this method, highlighting the efficiency of the ultrasonic method in shorter extraction times using polar solvents.

Alara et al. [10] and Annegowda et al. [22] emphasize the preference for shorter extraction times in UAE to preserve the integrity of phytochemicals. Da Silva et al. [23] and Ez Zoubi et al. [24] suggest that the use of a combination of two solvents, such as ethanol and water, promotes molecular interactions with phenolic compounds present in the plant species, contributing to the extraction process. The inclusion of water in all combinations is based on its high polarity and miscibility with organic solvents, which play a crucial role in enhancing the process efficacy [25].

In contrast, the Soxhlet‐extracted sample SBT4, obtained over 480 min with 70% ethanol, exhibited the highest phenolic content (385.95 ± 1.2 mg EAG g^−1^) for this method. The extended extraction time and higher ethanol content facilitated the solubilization and extraction of bioactive compounds. Oussaid et al. [26] corroborate the importance of appropriate extraction times to ensure the extraction of most active compounds using Soxhlet extraction. Furthermore, elevated temperatures, compared to room temperature, enhance solubility and diffusion properties of the solvent, thereby facilitating the release and extraction of substances such as phenolic compounds, which tend to bind to cellulose and hemicellulose in the cell wall structure [27, 28].

While both methods were effective in extracting phenolic compounds, extracts obtained by Soxhlet exhibited significantly higher levels in eight samples (SBT1, SBT2, SBT4, SBT6, SBT8, SBT9, SBT10, SBT11). The superiority of the method can be attributed to the specific operational conditions of the equipment used, which maintains a continuous flow of heated solvent over the plant matrix, promoting more efficient solvent penetration and enabling the extraction of less accessible phenolic compounds that might not be fully released by faster methods, such as UAE [28]. The samples UBT8 and SBT8 showed similar results in both yield and phenolic content, which will be explained by the chemical and structural characteristics of the plant matrix, associated with the use of the same solvent (78% ethanol). The high solubility of phenolic compounds in ethanol, possibly in their free form, and a lower structural complexity of the matrix seem to have favored an efficient extraction in both methods, minimizing differences in yield and content of phenolic compounds.

Regarding flavonoid content, values ranged from 19.22 to 137.83 mg ER g^−1^ in UAE extracts and from 17.83 to 73.66 mg ER g^−1^ in Soxhlet extracts. UAE demonstrated superior efficiency in flavonoid content, especially in sample UBT8 (137.83 ± 5.0 mg ER g^−1^) compared to sample SBT3 (73.66 ± 1.7 mg ER g^−1^), which were the highest values for each method. This result is likely due to the mechanical action of ultrasonic waves, which facilitate the rupture of plant cells and release of bioactive compounds [20]. It is worth noting that the flavonoid contents were significantly similar between samples UBT4 and SBT4, and between UBT11 and SBT11.

To date, no records in the literature have been found regarding extractions performed on *C. rubra* bark under the specific conditions of this study, limiting direct comparisons. However, the results obtained here were superior to those reported in similar studies on other plants of the same family (Lecythidaceae). Fontoura et al. [29] used a water bath extraction and investigated the influence of variables such as time, temperature, and ethanol content on total phenolic content in *Bertholletia excelsa* bark. They reported values ranging from 10.86 ± 0.007 to 20.44 ± 0.121 g EAG 100 g^−1^ under optimized extraction conditions.

Vasquez‐Rojas et al. [30] determined the phenolic content in *B. excelsa* bark using UBT, finding values of 5.435 mg EAG 100 g^−1^ with a water/methanol solution (50% v/v) and 4.673 mg EAG 100 g^−1^ with water/ethanol (50% v/v). While Vasquez‐Rojas et al. [30] applied a similar extraction method using polar solvents, experimental conditions such as temperature (*T* = 50°C) and sonication time (*t* = 20 min) differed from those evaluated in this study. These methodological variations may explain discrepancies in the results when comparing different species within the same family.

### 2.2 Response Surface Model

DCCR was employed to systematically investigate how extraction time (*X*_1_) and ethanol concentration (*X*_2_) influence the yield and concentrations of phenolic compounds and flavonoids in the extracts from the bark of *C. rubra*, obtained by both UAE and Soxhlet extraction. The primary objective was to identify the optimal conditions to maximize yield and the concentrations of bioactive compounds. The mathematical model used in this study was established with a 5% significance level.

**FIGURE 1:**
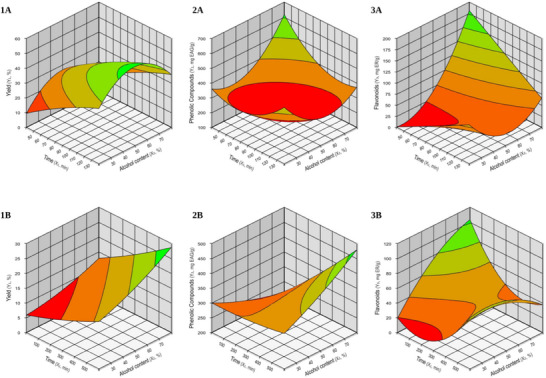
Response surface plots showing the effect of extraction time and ethanol concentration on: (1) Yield, (2) phenolic compound content, and (3) flavonoid content in extracts from *Cariniana rubra* bark obtained by (A) ultrasonic‐assisted extraction (UAE) and (B) Soxhlet apparatus.

The response surface plots, presented in Figure 1, illustrate the effects of extraction time and ethanol concentration on the yield, phenolic compound content, and flavonoid content of the *C. rubra* bark extracts.

The solvent choice and extraction duration, a crucial role in both the extraction process and its overall efficiency [31]. It is evident from Figure 1.1A that the yield in UAE increased proportionally with both ethanol concentration and extraction time, reaching a saturation point. The optimal conditions for high yield (indicated by the green region) are within the range of 120–140 min of extraction time and ethanol concentrations between 50% and 70%. The desirability analysis for yield, with a maximum value of 1.0, presented in Table 2, indicates that the optimal condition was achieved with an ethanol concentration of 50% and an extraction time of 132 min, resulting in a maximum yield of 49.60%.

**TABLE 2:** Desirability analysis for the optimization of yield (*Y*), phenolic compounds (PC), and flavonoid content (FC) of *Cariniana rubra* bark extracts obtained by ultrasound‐assisted extraction (UAE) and Soxhlet apparatus.

Desirability									
Ultrassom assisted					Soxhlet				
Extract	*Y*	PC	FC	D	Extract	*Y*	PC	FC	D
UBT1	0.04	0.00	0.15	0.00	SBT1	0.00	0.58	0.00	0.00
UBT2	0.75	0.67	0.26	0.50	SBT2	0.54	0.91	0.14	0.41
UBT3	0.09	0.86	0.67	0.38	SBT3	0.06	0.00	1.00	0.00
UBT4	0.77	0.73	0.10	0.38	SBT4	0.73	1.00	0.31	0.61
UBT5	0.17	1.00	0.06	0.22	SBT5	0.44	0.08	0.86	0.30
UBT6	1.00	0.03	0.18	0.18	SBT6	0.72	0.79	0.99	0.82
UBT7	0.08	0.74	0.00	0.00	SBT7	0.21	0.04	0.26	0.13
UBT8	0.00	0.86	1.00	0.00	SBT8	1.00	0.91	0.32	0.67
UBT9	0.72	0.02	0.08	0.11	SBT9	0.38	0.87	0.40	0.51
UBT10	0.66	0.04	0.11	0.14	SBT10	0.44	0.49	0.38	0.43
UBT11	0.68	0.14	0.14	0.23	SBT11	0.46	0.41	0.40	0.42

Abbreviations: D, global desirability; FC, flavonoid content; PC, phenolic compounds; SBT, Soxhlet bark testing; UBT, ultrasonic bark testing; Y, yield.

Model validation was performed by comparing experimental values with predicted values. The comparison shows that, for UAE, the predicted and experimental yields showed strong agreement, with small deviations. The highest yield (49.60%) obtained experimentally was close to the predicted value (51.77%), confirming the reliability of the model.

The analysis of variance (ANOVA) for the extraction yield, phenolic compound content, and total flavonoid content in the *C. rubra* bark extracts obtained by both UAE and Soxhlet apparatus are presented in Tables 3 and 4, respectively. The ANOVA is a reliable method for evaluating the quality of the fitted model, allowing the identification of the influence of various factors on the response and assessing the relevance of these influences [32].

**TABLE 3:** Analysis of variance (ANOVA) for yield, phenolic compounds, and total flavonoids in the *Cariniana rubra* bark extracts obtained by ultrasonic‐assisted extraction (UAE).

Ultrasonic assisted						
Variable	Variation source	Sum of squares	Degrees of freedom	Mean square	*F*_Value_	*F*_Table_
Yield	Regression	842.1	5	168.4	43.3	0.00041
	Residuals	19.5	5	3.9		
	Total	861.5	10			
Phenolic compounds	Regression	37 990.9	5	7598.2	2.0	0.22511
	Residuals	18 551.7	5	3710.3		
	Total	56 542.6	10			
Flavonoids	Regression	10 556.8	5	2111.4	4.0	0.07715
	Residuals	2638.5	5	527.7		
	Total	13 195.3	10			

**TABLE 4:** Analysis of variance (ANOVA) for yield, phenolic compounds, and total flavonoids in the *Cariniana rubra* bark extracts obtained by Soxhlet apparatus.

Soxhlet						
Variável	Variation source	Sum of squares	Degrees of freedom	Mean square	*F*_Value_	*F*_Table_
Yield	Regression	210.1	5	42.0	1.8	0.26887
	Residuals	117.3	5	23.5		
	Total	327.4	10			
Phenolic compounds	Regression	18 291.1	5	3658.2	2.0	0.23476
	Residuals	9210.8	5	1842.2		
	Total	27 501.9	10			
Flavonoids	Regression	2489.6	5	497.9	2.4	0.18248
	Residuals	1049.5	5	209.9		
	Total	3539.15	10			

Based on the data presented (Table 3), the model for yield showed significance only for the UAE (*p* = 0.0041), with an *R* ^2^ of 97.74%, indicating that the model explains a substantial proportion of the variation in extraction yield [32, 33]. The equation resulting from the ANOVA for this response $\left(Y=41.85+7.99X_{1}-0.69X_{1}^{2}-0.14X_{2}-7.49X_{2}^{2}-0.21X_{1}X_{2}\right)$, a quadratic model encompassing all possible variables, allows for the calculation of extraction conditions beyond those tested in this experiment.

Pareto analysis (Figure 2.1A) for the yield obtained via UAE reveals that the mean, representing the overall combined impact of all variables on the response, has the largest effect on yield, followed by extraction time (*X*_1_) and ethanol concentration (*X*_2_).

**FIGURE 2:**
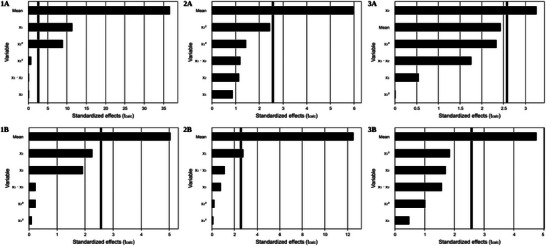
Pareto diagrams of (1) yield, (2) phenolic compound content, and (3) flavonoid content of *Cariniana rubra* bark extracts obtained by (A) ultrasonic‐assisted extraction (UAE) and (B) Soxhlet apparatus, where *X*_1_ corresponds to time and *X*_2_ to ethanol concentration.

Ullah et al. [34], in their study on the optimization of UAE from Gemlik olive fruit, reported that extraction time (30 min) and ethanol concentration (75%) were significant variables for maximizing extract yield. The differences between their findings and those of the present study may be attributed to the characteristics of the plant matrix, which influence the extraction process.

The linear effect of time (*X*_1_) and the quadratic effect of ethanol content (*X*_2_) had a significant impact on the yield response, suggesting the existence of optimal or maximum points that should be considered when adjusting these parameters. This indicates that the more rigid structure of the bark requires a longer extraction time to allow the significant release of the bioactive compounds present.

The Pareto diagram (Figure 2.1B) for Soxhlet yield indicates that, although the variables do not have a statistically significant impact on the yield, time (X_1_) shows the largest effect. To maximize the yield in this case, it would be prudent to adjust the extraction time to values close to the identified optimal range (300–600 min) and consider solvent concentrations above 60%.

These data, supported by the response surface analysis, indicate that UAE demonstrated greater efficiency for yield compared to Soxhlet extraction, suggesting its superiority in terms of extraction efficiency.

Similarly to what was observed for the yield in Soxhlet extraction, the response surface models for phenolic compounds extracted by UAE (Figure 1.2A) and Soxhlet (Figure 1.2B), as well as for flavonoids extracted by UAE (Figure 1.3A) and Soxhlet (Figure 1.3B), did not show statistical significance (*p* > 0.05) (Tables 3 and 4). This suggests that the independent variables (time and ethanol concentration) for phenolic content (Figure 2.2A,2B) and flavonoids (Figure 2.3A,2B) do not significantly explain the variation in the response. This implies that changes in these variables do not have a statistically relevant effect on the amount of phenolic compounds and flavonoids extracted.

For flavonoids, *R* ^2^ values were relatively high (80% for UAE and 70.35% for Soxhlet extraction), indicating that, despite the lack of statistical significance (Tables 3 and 4), the model variables explain a substantial portion of the variation in the response.

The desirability analysis identified the optimal conditions for each extraction method, corresponding to 90 min and 78% ethanol for UAE, and 120 min with 70% ethanol for Soxhlet (Table 2). The experimental and predicted values for UAE were 137.83 and 115.02 mg RE g^−1^, respectively, while for Soxhlet, the values were 73.66 mg RE g^−1^ (experimental) and 67.76 mg RE g^−1^ (predicted).

For phenolic compounds, *R* ^2^ values were moderate (67.19% for UAE and 66.51% for Soxhlet extraction), but equally not significant (Tables 3 and 4). The optimal conditions were determined as 48 min and 50% ethanol for UAE, and 480 min and 70% ethanol for Soxhlet extraction (Table 2). The experimental and predicted values for UAE were 382.62 and 312.20 mg GAE g^−1^, respectively. For the Soxhlet technique, the experimental values were 385.95 mg GAE g^−1^ and the predicted values were 397.58 mg GAE g^−1^.

The desirability analysis determined the optimal extraction conditions for both methods, with a maximum desirability index of 1 (Table 2). However, the discrepancies between the experimental and predicted values for flavonoids and phenolic compounds suggest that the predictive model needs to be improved. This could lead to a more effective optimization of the extraction conditions, particularly for UAE, where the prediction was less accurate.

The analysis of global desirability (*D*) identified the most suitable conditions for simultaneously maximizing the three evaluated responses [35]. In UAE, extract UBT2 exhibited the best condition with 120 min and 70% ethanol (*D* = 0.50). In the Soxhlet method, extract SBT6 achieved the highest desirability value (*D* = 0.82), indicating optimal conditions at 555 min and 50% ethanol. Since *D* values close to 1 reflect greater suitability of the experimental conditions for simultaneously meeting the desired responses, the Soxhlet method demonstrated the best overall performance.

Although the individual optimization of phenolic compounds and flavonoids was not statistically significant, the desirability analysis provides valuable insights for process improvement, ensuring a balanced approach among the investigated parameters. Furthermore, while RSM is a powerful optimization tool, adjustments to both the model and the experimental approach are recommended to enhance prediction accuracy and achieve more statistically robust results.

### 2.3 Evaluation of Antioxidant Potential

In the present study, DPPH•, ABTS•^+^, and ferric reducing antioxidant power (FRAP) assays were employed to assess the antioxidant activity of *C. rubra* extracts obtained by both UAE and Soxhlet extraction, as presented in Table 5.

**TABLE 5:** Results of the antioxidant activity evaluation by DPPH• (µg mL^−1^), ABTS•^+^ (Trolox g^−1^), and FRAP (µM g^−1^ SF) assays for *Cariniana rubra* bark extracts obtained by ultrasonic‐assisted extraction (UAE) and Soxhlet apparatus.

Ultrasonic assisted				Soxhlet			
Extract	DPPH• (IC_50_) (µg mL^−1^)	ABTS•^+^ (µM Trolox g^−1^)	FRAP (µM g^−1^ FS)	Extract	DPPH• (IC_50_) (µg mL^−1^)	ABTS•^+^ (µM Trolox g^−1^)	FRAP (µM g^−1^ FS)
UBT1	30.29 ± 1.38^c^	28.78 ± 0.20^e^	379.00 ± 6.67^d^	SBT1	18.44 ± 1.22^e^	12.00 ± 2.7^d^	299.00 ± 5.00^e^
UBT2	37.73 ± 0.42^a^	42.00 ± 4.70^c^	198.44 ± 7.52^g^	SBT2	28.36 ± 1.41^c^	22.33 ± 4.7^b^	517.33 ± 6.67^b^
UBT3	30.89 ± 1.78^c^	45.56 ± 1.70^b^	295.67 ± 6.01^f^	SBT3	37.00 ± 0.61^a^	13.78 ± 7.2^d^	273.44 ± 5.85^f^
UBT4	34.08 ± 0.66^b^	43.78 ± 3.20^b^	392.33 ± 6.67^c^	SBT4	17.08 ± 1.73^e^	19.11 ± 2.5^c^	365.11 ± 5.85^c^
UBT5	20.06 ± 0.63^f^	53.11 ± 3.80^a^	205.67 ± 5.00^g^	SBT5	13.77 ± 1.71^f^	10.11 ± 5.6^d^	216.78 ± 2.55^g^
UBT6	35.14 ± 0.52^b^	53.67 ± 1.70^a^	313.44 ± 7.52^e^	SBT6	23.21 ± 1.45^d^	23.33 ± 6.4^b^	315.67 ± 6.67^d^
UBT7	32.11 ± 0.22^c^	37.33 ± 3.70^d^	419.56 ± 5.85^b^	SBT7	33.60 ± 1.44^b^	3.78 ± 2.3^e^	558.44 ± 5.09^a^
UBT8	26.26 ± 0.40^d^	33.11 ± 1.50^d^	453.44 ± 2.55^a^	SBT8	36.20 ± 1.04^a^	38.67 ± 3.8^a^	554.00 ± 5.00^a^
UBT9	21.88 ± 1.10^e^	20.00± 2.60^f^	322.33 ± 6.01^e^	SBT9	22.60 ± 0.87^d^	11.78 ± 3.7^d^	211.78 ± 5.85^g^
UBT10	22.64 ± 0.26^e^	21.78 ± 1.40^f^	319.56 ± 6.31^e^	SBT10	23.02 ± 0.07^d^	11.78 ± 1.8^d^	210.11 ± 3.47^g^
UBT11	20.82 ± 1.97^f^	20.44 ± 2.20^f^	320.67 ± 3.33^e^	SBT11	22.64 ± 0.44^d^	12.56 ± 3.4^d^	211.22 ± 7.70^g^
Trolox	23.64 ± 1.63^d^	—	—		23.64 ± 1.63^d^	—	—

*Note*: Values represent the mean ± SD (*n* = 3). Different superscripted lowercase letters in the rows indicate significant differences (*p* < 0.05, ANOVA followed by Tukey's test).

Abbreviations: FS, ferrous sulfate; SBT, Soxhlet bark testing; SD, standard deviation; UBT, ultrasonic bark testing.

The results indicate a significant antioxidant capacity in the *C. rubra* extracts. In the DPPH• assay, a lower IC_50_ concentration reflects a higher antioxidant capacity of the extract, as it indicates that a smaller quantity of the extract is required to reduce the DPPH• radical concentration by 50%. Therefore, IC_50_ values lower than the reference standard indicate elevated antioxidant activity [36]. In the DPPH• assay, the extracts UBT5 (20.06 ± 0.63 µg mL^−1^), UBT11 (20.82 ± 1.97 µg mL^−1^), SBT5 (13.77 ± 1.71 µg mL^−1^), SBT1 (18.44 ± 1.22 µg mL^−1^), and SBT4 (17.08 ± 1.73 µg mL^−1^) stood out, surpassing the antioxidant capacity of the Trolox standard (23.64 ± 1.63 µg mL^−1^). Lima Neto et al. [37] found IC_50_ values of 9.31 ± µg mL^−1^, higher than the caffeic acid standard (1.89 ± µg mL^−1^), in *C. rubra* bark extracts obtained by maceration in 90% ethanol. Silva et al. [5] obtained lower values (0.44 µg mL^−1^) compared to the ascorbic acid standard (1.9 ± µg mL^−1^) in *C. rubra* bark extracts obtained by maceration in a 70% hydroethanolic solution.

The variations in IC_50_ values across studies can be attributed to several methodological factors, including differences in extraction efficiency, solvent water content, and the concentration and stability of the extracted antioxidant compounds.

In addition, the soil and climate conditions at the plant collection sites play a pivotal role in determining various plant characteristics. Abiotic environmental stresses, such as extreme temperatures and drought, can alter plant metabolism, leading to either inhibition or enhancement of secondary metabolite production, which is crucial for species defense and adaptation [38].

In the 2,2′‐azinobis(3‐ethylbenzothiazoline‐6‐sulfonic acid) (ABTS) and FRAP assays, higher antioxidant activities were observed in the extracts with higher concentrations. For the ABTS•^+^ assay, the ultrasonically extracted samples UBT5 (53.11 ± 3.80 µM Trolox g^−1^) and UBT6 (53.67 ± 1.70 µM Trolox g^−1^) exhibited the highest antioxidant activity, while among the Soxhlet extracts, only SBT8 stood out (38.67 ± 3.80 µM Trolox g^−1^). In the FRAP assay, the ability of *C. rubra* extracts to reduce Fe^3+^ to Fe^2+^ was significantly greater in the ultrasonically extracted sample UBT8 (453.44 ± 2.55 µM SF g^−1^), as well as in Soxhlet samples SBT7 (558.44 ± 5.09 µM SF g^−1^) and SBT8 (554.00 ± 5.00 µM SF g^−1^). These results underscore the considerable antioxidant potential of *C. rubra*, as evidenced through different extraction methods and benchmark parameters.

Silva et al. [5] similarly reported the antioxidant activity of *C. rubra* bark using 2,2‐diphenyl‐1‐picrylhydrazyl (DPPH) (0.44 ± 0.16 µg mL^−1^; ascorbic acid standard 1.9 ± 0.01) and FRAP assays (64.00 ± 4.43 µM SF g^−1^; ascorbic acid standard 1.68 ± 0.02). Comparable antioxidant activities have been observed in other species from the Lecythidaceae family, such as *B. excelsa* and *Barringtonia racemosa* (L.) Spreng, as investigated by Fontoura et al. [29] and Kong et al. [39], respectively, using DPPH and ABTS assays.

The extracts analyzed in this study exhibited high levels of phenolic compounds, which are likely responsible for the substantial antioxidant capacities observed in *C. rubra* extracts. The bioactivity of these compounds is primarily attributed to the number and configuration of hydroxyl groups in their structures, as well as the associated chemical substituents [40, 41, 42]. The ability of these groups to donate electrons or hydrogen atoms effectively neutralizes free radicals and enhances antioxidant activity [43, 44, 45].

### 2.4 Fourier‐Transform Infrared Analysis

**FIGURE 3:**
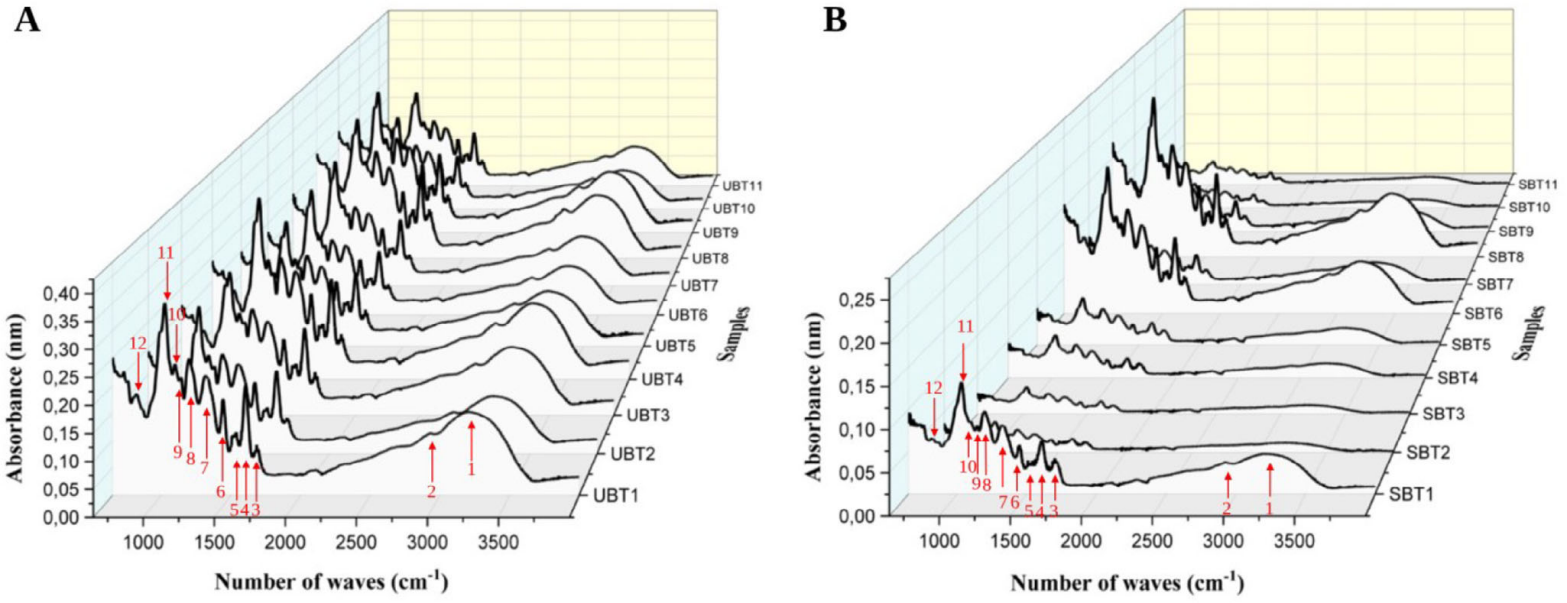
FT‐IR spectra of *Cariniana rubra* bark extract obtained through (A) ultrasound‐assisted extraction (UAE) and (B) Soxhlet apparatus.

The results of the Fourier‐Transform Infrared (FT‐IR) spectroscopic analysis of *C. rubra* extracts are presented in Figure 3 and Table 6. The peaks in the FT‐IR spectra (indicated by red numbered arrows) correspond to the presence of various functional groups.

**TABLE 6:** Assignments of FT‐IR spectral wavenumbers for *Cariniana rubra* extracts obtained via ultrasound‐assisted extraction (UAE) and (B) Soxhlet apparatus.

Peak	Wavelength (cm^−1^)^a^	Associated functional group
1	3200–3245	Stretching ─OH [46, 47, 48]
2	2916–2941	Stretching ─CH2, ─CH3 [21, 46, 49]
3	1684–1701	Stretching C═C [46, 48, 51]
4	1602–1610	Stretching C═C (aromatic) [21, 47, 49]
5	1533–1541	Stretching C═C (aromatic) [46, 49]
6	1437–1448	Stretching C═C (aromatic) [46, 49]
7	1314–1331	Stretching C─O (aromatic) [49]
8	1195–1209	Stretching C─C and C─O [21, 46]
9	1136–1149	Stretching C─C and C─O [21, 46]
10	1095–1104	Stretching C─C and C─O [46]
11	1022–1031	Stretching C─C and C─O [46, 49]
12	814–827	Bending ═C─H out of plane [46, 49]

^a^ Range of variation among the extracts.

The stretching vibrations of the ─OH groups, present in alcohols and phenols, are characterized by oscillatory peaks ranging from 3200 to 3245 cm^−1^ [46, 47, 48]. The bands in the range of 2916–2941 cm^−1^ are attributed to the stretching vibrations of the ─CH_2_ and ─CH_3_ groups [21, 47, 49]. The phenolic signal can be found in the 1680–900 cm^−1^ region [50].

The weak absorption peak observed between 1684 and 1701 cm^−1^ results from the stretching of C═C bonds in aromatic rings [46, 48, 51] corroborated by the weak absorption between 814 and 827 cm^−1^ [46]. Three bands of variable intensity, indicative of C═C stretching vibrations in the aromatic ring, were identified within the ranges of 1602–1610, 1533–1541, and 1437–1448 cm^−1^ [21, 46, 49].

Aromatic C─O bond stretching is evidenced in the region between 1314 and 1149 cm^−1^ [21, 46, 49]. The distinct bands between 1095 and 1031 cm^−1^ may be associated with the deformation of C─O bonds in secondary alcohols, as well as vibrations of C─H bonds in side chains [46].

The vibrational bands between 814 and 827 cm^−1^ represent the bending of ═C─H bonds, resulting in an out‐of‐plane arrangement of the molecule, which implicates the three‐dimensional structure and chemical properties of the molecule. The functional groups identified in the *C. rubra* extracts, such as hydroxyl (─OH), methyl (CH) groups, and carbon–carbon double bonds (C═C), are associated with phenolic compounds, including flavonoids [10, 52, 53]. These groups were detected in all analyzed extracts, with variations in peak intensities that reflect differences in the relative concentrations of these phenolic compounds in each extract, corroborating the results obtained in the quantification of these compounds and the analysis of the antioxidant activity of *C. rubra*.

### 2.5 Liquid Chromatography With Diode Array Detection Analysis

**FIGURE 4:**
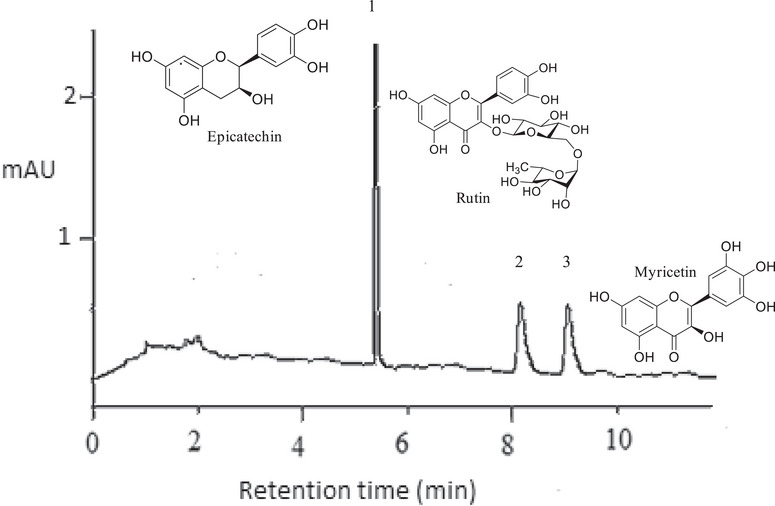
Representative chromatogram (LC–DAD) of the *Cariniana rubra* bark extracts. Peak 1: epicatechin; Peak 2: rutin; Peak 3: myricetin.

The results from the liquid chromatography with diode array detection (LC–DAD) chromatographic analysis are presented in Table 7. The chromatograms of all samples displayed identical profiles across both methodologies, as illustrated in Figure 4, with variations in the concentrations of each detected metabolite (Table 7).

**TABLE 7:** Quantification of chemical compounds using LC–DAD in extracts from *Cariniana rubra* bark (mg g^−1^) obtained by ultrasound‐assisted extraction (UAE) and Soxhlet extraction apparatus.

UAE				Soxhlet			
Extracts	Compounds (mg g^−1^)			Extracts	Compounds (mg g^−1^)		
	Epicatechin	Rutin	Myricetin		Epicatechin	Rutin	Myricetin
UBT1	67.50 ± 0.20^h^	9.90 ± 0.12^e^	9.4 ± 0.10^g^	SBT1	55.50 ± 0.10^g^	10.90 ± 0.06^e^	9.80 ± 0.10^f^
UBT2	68.00 ± 0.10^g^	10.10 ± 0.06	9.7 ± 0.00^f^	SBT2	67.30 ± 0.06^a^	13.10 ± 0.10^a^	11.5 ± 0.06^a^
UBT3	73.80 ± 0.00^c^	11.50 ± 0.06^c^	11.1 ± 0.00^b^	SBT3	53.90 ± 0.10^h^	10.60 ± 0.06^f^	8.70 ± 0.10^g^
UBT4	74.30 ± 0.15^b^	11.80 ± 0.06^b^	11.4 ± 0.15^a^	SBT4	59.90 ± 0.10^d^	12.00 ± 0.06^c^	10.50 ± 0.10^d^
UBT5	69.40 ± 0.15^f^	9.90 ± 0.10^e^	10.1 ± 0.00^e^	SBT5	50.70 ± 0.12^i^	9.70 ± 0.06^g^	7.80 ± 0.12^h^
UBT6	71.10 ± 0.00^d^	11.20 ± 0.10^d^	10.6 ± 0.06^c^	SBT6	60.10 ± 0.06^c^	12.30 ± 0.06^b^	10.80 ± 0.10^c^
UBT7	65.90 ± 0.15^i^	9.30 ± 0.10^f^	9.1 ± 0.00^h^	SBT7	63.40 ± 0.06^b^	13.00 ± 0.06^a^	11.00 ± 0.06^b^
UBT8	78.60 ± 0.15^a^	13.50 ± 0.10^a^	11.3 ± 0.06^a^	SBT8	59.60 ± 0.15^e^	11.70 ± 0.06^d^	10.30 ± 0.06^e^
UBT9	70.90 ± 0.06^e^	11.00 ± 0.10^d^	10.4 ± 0.06^d^	SBT9	59.00 ± 0.10^f^	11.20 ± 0.06^d^	10.10 ± 0.06^e^
UBT10	70.90 ± 0.06^e^	11.00 ± 0.10^d^	10.4 ± 0.06^d^	SBT10	59.00 ± 0.10^f^	11.20 ± 0.06^d^	10.10 ± 0.06^e^
UBT11	70.90 ± 0.06^e^	11.00 ± 0.10^d^	10.4 ± 0.06^d^	SBT11	59.00 ± 0.10^f^	11.20 ± 0.06^d^	10.10 ± 0.06^e^

*Note*: Values represent the mean ± SD (*n* = 3). Different lowercase superscript letters between the rows indicate significant differences (*p* < 0.05, ANOVA followed by Tukey's test).

Abbreviations: SBT: Soxhlet bark testing; SD, standard deviation; UBT, ultrasonic bark testing.

In the extracts from the bark of *C. rubra* obtained via UAE, the presence of epicatechin was detected with a retention time (RT) of 5.11 min, and concentrations ranging from 65.90 to 78.60 mg g^−1^ of extract; rutin, with an RT of 8.13 min, and concentrations ranging from 9.30 to 11.80 mg g^−1^ of extract; and myricetin, with an RT of 9.19 min, and concentrations ranging from 9.10 to 11.40 mg g^−1^ of extract. For the extracts obtained via Soxhlet, the concentration ranges were 50.70–67.30 mg g^−1^ (RT = 5.13 min) for epicatechin, 9.70–13.10 mg g^−1^ (RT = 8.14 min) for rutin, and 7.80–11.50 mg g^−1^ (RT = 9.19 min) for myricetin.

The highest concentration of phenolic compounds tends to be associated with higher levels of specific compounds. This pattern is evidenced in the sample UBT8, which exhibited the highest flavonoid concentration (137.83 mg ER g^−1^), correlating with the elevated levels of epicatechin, rutin, and quercetin detected by LC–DAD, as well as the antioxidant capacity confirmed by DPPH, ABTS, and FRAP assays. However, this correlation is not absolute, as the composition of extracts can vary depending on the extraction methods and conditions used.

Tukey's test showed that the UBT8 (78.60 ± 0.15 mg g^−1^) and SBT2 (67.30 ± 0.06 mg g^−1^) samples exhibited the highest concentrations of epicatechin, surpassing other samples such as UBT1 (67.50 ± 0.20 mg g^−1^) and SBT5 (50.70 ± 0.12 mg g^−1^). The high extraction efficiency observed in UBT8 is attributed to the combination of a high ethanol concentration (78%) with a 90‐min extraction time, which is consistent with the results obtained in antioxidant assays. Similarly, the SBT2 sample demonstrated that prolonged extraction times (480 min) can compensate for a lower ethanol concentration (30%), resulting in significant epicatechin concentrations. Epicatechin, belonging to the flavonol class, possesses antioxidant, anti‐inflammatory properties, enhances muscle performance, and may contribute to the prevention of cardiovascular and cerebrovascular diseases [54].

For rutin, the UBT8 sample, extracted with 78% ethanol and 90 min of extraction, exhibited the highest concentration (13.50 ± 0.10 mg g^−1^), supporting the antioxidant activity observed in the DPPH, ABTS, and FRAP assays. Notably, the SBT7 sample (13.00 ± 0.06 mg g^−1^), obtained with a lower ethanol concentration (22%) and a longer extraction time (300 min), also achieved high concentrations, indicating that prolonged extraction times can be effective for extracting this compound. The flavonoid rutin exhibits anti‐inflammatory, antioxidant, antiangiogenic, and antitumor activities, being effective in preventing metabolic reprogramming and restoring lipid metabolism [55, 56].

Regarding quercetin, the UBT4 sample (11.4 ± 0.15 mg g^−1^) recorded the highest concentration, with 70% ethanol and 120 min of extraction, highlighting the importance of an adequate extraction time for maximizing yield. The SBT2 sample again stood out with a high concentration of quercetin (11.5 ± 0.06 mg g^−1^), reinforcing the crucial role of prolonged extraction time. Quercetin demonstrates anti‐inflammatory, antioxidant, antibacterial, antitumor, antiviral, cardiovascular, neuroprotective, and hepatoprotective effects [41, 57].

Overall, higher ethanol concentrations (70%–78%) and moderate extraction times (60–120 min) were more effective for extracting epicatechin, rutin, and quercetin. However, in some samples, prolonged extraction times, such as in SBT2, also resulted in high concentrations, even with lower ethanol concentrations.

The positive correlation between phenolic content and antioxidant capacity is well established and is supported by the results obtained in this study.

## 3 Conclusions

This study evaluated the efficacy of UAE and Soxhlet extraction for isolating bioactive compounds from the bark of *C. rubra*. RSM revealed that optimization was statistically significant only for the yield of UAE (*p* = 0.0041). The UAE achieved a maximum yield of 49.60% when using a 50% ethanol concentration and a 132‐min extraction time.

The total phenolic content (385.95 mg EAG g^−1^) and flavonoid content (137.83 mg ER g^−1^) obtained in this study were notably higher than those reported in the literature for *C. rubra* and related plant species, highlighting the effectiveness of the applied optimization process.

The extracts demonstrated high antioxidant capacity, as evidenced by DPPH, ABTS, and FRAP assays, directly correlated with the high concentrations of bioactive compounds such as epicatechin, rutin, and myricetin, identified by LC–DAD. These findings suggest that *C. rubra* bark has significant potential for applications in the natural product and pharmaceutical industries.

Although statistical significance (*p* > 0.05) was not observed for the optimization of phenolic and flavonoid contents or for the extraction yield using Soxhlet extraction, the established models effectively explained a substantial portion of the data variability. These results indicate that while further adjustments may be necessary to optimize extraction efficiency, the methodologies employed have already provided promising outcomes.

## 4 Experimental Section

### 4.1 Plant Material Collection

The bark of *C. rubra* was collected in the municipality of Pium, Tocantins (TO), specifically at the Canguçu Private Natural Heritage Reserve (RPPN Canguçu), located at 9°58′47″ S and 50°2′12″ W, within the transition zone between the Amazon and Cerrado biomes, on October 15, 2021, around 2:00 p.m. The bark samples were cataloged and incorporated into the herbarium collection of the Tocantins State University (HUTO), located at the Tocantins State University (UNITINS) in Palmas, TO, under the voucher number HUTO 8161 and registered in SisGen under the number AE8F6D0. The bark samples were dried in an oven at 60°C for 48 h, ground using a Willey knife mill (Star FT 50, Fortinox, Piracicaba, Brazil), and stored in glass containers to prevent moisture exposure.

### 4.2 Reagents

All solvents and chemical reagents used in this study are of analytical grade. The reagents Folin–Ciocalteu phenol, DPPH, ABTS, 2,4,6‐tris(2‐pyridyl)‐s‐triazine (TPTZ), the standards rutin and Trolox, as well as analytical standards for LC–DAD, including gallic acid, protocatechuic acid, chlorogenic acid, epicatechin, rutin, myricetin, and kaempferol, were purchased from Sigma‐Aldrich (Cotia, São Paulo, Brazil). Ferrous sulfate was supplied by ACS Científica (Sumaré, São Paulo, Brazil), while gallic acid and ferric chloride were obtained from Dinâmica (Catanduva, São Paulo, Brazil). Solvents such as methanol, ethanol, absolute methanol, glacial acetic acid, pyridine, and potassium persulfate were acquired from Neon (Suzano, São Paulo, Brazil). Sodium carbonate was supplied by Anidrol (Diadema, São Paulo, Brazil), aluminum chloride by Cromoline (Diadema, São Paulo, Brazil), and sodium acetate by Isofar (Duque de Caxias, Rio Grande do Sul, Brazil).

### 4.3 Experimental Design

The experimental design was performed using the Protimiza Experimental Design software. A central composite rotatable design (CCRD) with two levels and two factors (2^2^) was chosen, incorporating three central points and four axial points. This design was used to evaluate the combined effects of the independent variables, time (*X*_1_) and ethanol concentration (*X*_2_), coded at different levels (−1 and +1), with a central point (0) and two axial points (−1.41 and +1.41), leading to a total of 11 experimental runs (Table 8). The yield determination was conducted once, while the other experiments were randomized and performed in triplicate to minimize variability effects on the observed responses. This design was applied to both extraction methods: UAE and Soxhlet extraction, each considering the technical limitations of the respective equipment.

**TABLE 8:** Real and coded values for the combination of time (min) and ethanol concentration (%) used in the preparation of extracts from the bark of *Cariniana rubra* by ultrasound‐assisted extraction (UAE) and Soxhlet extraction.

	Independent variables			
	(*X*_1_) Time (min)		(*X*_2_) Ethanol concentration (%)	
Sample	Coded values	Real values	Coded values	Real values
Ultrassom assisted				
UBT1	−1	60	−1	30
UBT2	1	120	−1	30
UBT3	−1	60	1	70
UBT4	1	120	1	70
UBT5	−1.41	48	0	50
UBT6	+1.41	132	0	50
UBT7	0	90	−1.41	22
UBT8	0	90	+1.41	78
UBT9	0	90	0	50
UBT10	0	90	0	50
UBT11	0	90	0	50
Soxhlet				
SBT1	−1	120	−1	30
SBT2	1	480	−1	30
SBT3	−1	120	1	70
SBT4	1	480	1	70
SBT5	−1.41	45	0	50
SBT6	+1.41	555	0	50
SBT7	0	300	−1.41	22
SBT8	0	300	+1.41	78
SBT9	0	300	0	50
SBT10	0	300	0	50
SBT11	0	300	0	50

### 4.4 Preparation of Extracts

Two extraction methods were employed to obtain dry extracts from the bark of *C. rubra*. UAE was performed using a Q5.9/40A ultrasonic bath (Eco‐Sonics, Indaiatuba, Brazil), operating at a frequency of 40 kHz with an ultrasonic power of 200 W at 30°C. For the extraction process, solutions containing 4 g of the powdered sample and 200 mL of hydroethanolic solution were prepared, with ethanol concentrations of 22%, 30%, 50%, 70%, and 78%. Ethanol was selected as the solvent due to its chemical versatility and alignment with green chemistry principles. The extraction times followed the experimental design (Table 8). For extractions longer than 60 min (90, 120, and 132 min), the solvent was replaced with a fresh 200 mL volume every 60 min until the total extraction time was completed, and the supernatants were combined. The samples obtained in this assay were labeled as ultrasonic bark testing (UBT).

The Soxhlet extraction was performed using a Soxhlet apparatus (MA044/8/50, Marconi, Piracicaba, Brazil) at 70°C for the durations of 58, 120, 270, 420, and 482 min, as determined by the proposed experimental model (Table 8). The samples from this assay were labeled as SBT (Soxhlet Bark Testing).

Following the extraction processes, the solvent was removed using a rotary evaporator (FISATOM, São Paulo, Brazil) under a pressure of 600 mmHg and at a temperature of 50°C. The extracts were subsequently frozen at −70°C for 48 h and then lyophilized using a lyophilizer (LIOTOP L101, São Carlos, Brazil) for 96 h. The yield was calculated using Equation (1):

(1) $$R\left(\%\right)=\left(\mathrm{DE}\right)/\mathrm{BW}\times 100$$

where *R* is the yield (%), DE is the dry extract weight (g), and BW is the bark weight before extraction (g).

### 4.5 Quantification of Metabolites

#### 4.5.1 Quantification of Phenolic Compounds

The total phenolic content in the extracts was determined using the Folin–Ciocalteu colorimetric method, as described by Amorim et al. [58]. The details of the methodology are in the Supporting Information. A standard gallic acid calibration curve (*y* = 0.0013*x* + 0.0346; *R* ^2^ = 0.9933) was used, with concentrations ranging from 10 to 400 µg mL^−1^. All measurements were performed in triplicate, and the results were expressed as gallic acid equivalents per gram of lyophilized extract (mg EAG g^−1^).

#### 4.5.2 Quantification of Flavonoid Content

The total flavonoid content was determined using the colorimetric method described by Peixoto Sobrinho et al. [59], with modifications adapted from Soares et al. [60]. The details of the methodology are in the Supporting Information. A standard calibration curve of rutin (*y* = 0.0012*x* + 0.0016; *R* ^2^ = 0.9941) was employed for quantification, with concentrations ranging from 10 to 400 µg mL^−1^. The assays were conducted in triplicate, and the results were expressed as milligrams of rutin equivalents (RE) per gram of lyophilized extract (mg RE g^−1^).

#### 4.5.3 Evaluation of Antioxidant Potential

For the analysis of antioxidant activity, three methods were used (i) the inhibition of the stable free radical 2,2‐diphenyl‐1‐picrylhydrazyl (DPPH•), as described by Brand‐Williams et al. [61], with adaptations by Peixoto‐Sobrinho et al. [62]; (ii) the scavenging of the 2,2′‐azinobis‐3‐ethylbenzothiazoline‐6‐sulfonic acid radical (ABTS•^+^), following the method outlined by Rufino et al. [63]; and (iii) the FRAP, as described by Rufino et al. [64]. The details of the methodology are in the Supporting Information.

#### 4.5.4 LC–DAD Analysis

The analysis of *C. rubra* bark extracts obtained by UAE and Soxhlet extraction was conducted using LC–DAD. The extracts were solubilized in a 1:1 (v:v) mixture of water: methanol and evaluated on a Shimadzu LC‐20A Prominence liquid chromatograph (Shimadzu, Japan), equipped with a diode array detector (DAD), employing a C18 column (2.1 mm × 100 mm, 1.8 µm). The flow rate was set at 0.25 mL min^−1^, the injection volume was 2 µL, and the samples were maintained at 30°C during analysis.

A binary mobile phase with a gradient program was employed, combining Solvent A (water with 0.05% formic acid) and Solvent B (methanol) as follows: 85% A (0 min), 85%–60% A (3 min), 60%–55% A (3 min), 55%–30% A (2 min), 30%–55% A (2 min), 55%–85% A (1 min), and 85%–100% A (1 min).

The UV detection wavelength was adjusted to the maximum absorbance for the analytes. Identification was based on the comparison of RTs and DAD spectra of the sample peaks with those of standards. For quantification, standard calibration curves were prepared using the following analytical standards: gallic acid (*λ* = 271 nm; *R* ^2^ = 0.9960), protocatechuic acid (*λ* = 259 nm; *R* ^2^ = 0.9980), chlorogenic acid (*λ* = 326 nm; *R* ^2^ = 0.9990), epicatechin (*λ* = 278 nm; *R* ^2^ = 0.9940), rutin (*λ* = 354 nm; *R* ^2^ = 0.9940), myricetin (*λ* = 372 nm; *R* ^2^ = 0.9980), and kaempferol (*λ* = 363 nm; *R* ^2^ = 0.9960), in concentrations ranging from 0.10 to 10 µg mL^−1^ using external calibration. All standards were obtained from Sigma (Sigma, ≥ 98%, St. Louis, MO, USA).

#### 4.5.5 FT‐IR Analysis

FT‐IR spectroscopy was employed to identify the functional groups present in the different extracts. The analysis was conducted using a Cary 630 FTIR spectrophotometer (Agilent Technologies, Santa Clara, USA). The spectra were recorded in the range of 600–3500 cm^−1^, with a step size of 2 cm^−1^ and 32 scans per sample. The analysis was performed on the dried extracts.

### 4.6 Statistical Analysis

The results obtained for the quantification of phenolic and flavonoid compounds, as well as the determination of the antioxidant activity of the extracts (DPPH•, ABTS•^+^, and FRAP), were organized and subjected to descriptive analysis (mean and standard deviation) using Microsoft Excel (Microsoft Corporation, Redmond, WA, USA), version 16.66.1. One‐way ANOVA, followed by Tukey's post‐hoc test (*p* < 0.05), was performed using Sisvar software version 5.6. All assays were conducted in triplicate, and the results are expressed as mean ± standard deviation.

For the RSM analysis, statistical analysis, model prediction, and model equation validation were performed using Protimiza Experimental Design software (PROTIMIZA, 2014). Differences between groups were evaluated using ANOVA. Results are presented as mean ± standard deviation, and a *p* value below 0.05 was considered statistically significant.

## Author Contributions

**Aline Biggi Maciel**: conceptualization, data curation, formal analysis, investigation, visualization, writing – original draft, writing – review and editing. **Elis Ramos de Queiroz Jácome**: data investigation. **Naísa Andrade da Silva**: data investigation. **Rachel de Moura Nunes Fernandes**: formal analysis, investigation, project administration, writing – review and editing. **Claudia Andrea Lima Cardoso**: investigation, formal analysis. **Elisandra Scapin**: data curation, formal analysis, funding acquisition, project administration, resources, visualization, writing – review and editing.

## Conflicts of Interest

The authors declare no conflict of interest.

## Supporting information




**Supporting File 1**: cbdv70224‐sup‐0001‐SuppMat.pdf

## Data Availability

The data supporting the findings of this study are available from the corresponding author upon reasonable request.
