# Supplementary material for: Efficient Extraction of Phenolic Compounds From Cariniana rubra Gardener Ex Miers From the Legal Amazon: Ultrasound‐Assisted Optimization, Chemical Characterization, and Antioxidant Potential
Source: Chem Biodivers. 2025 Jul 17;22(11):e03256. doi: 10.1002/cbdv.202403256 (PMC12629157; doi:10.1002/cbdv.202403256)
Supplement: Supplementary file 1 — Supporting File 1: cbdv70224‐sup‐0001‐SuppMat.pdf [file CBDV-22-e03256-s001.pdf]

Supporting Information

Experimental Section

Quantification of Metabolites

Quantification of Phenolic Compounds

The total phenolic content in the extracts was determined using the Folin-Ciocalteu colorimetric method, as described by Amorim et al.[53]. Briefly, 0.5 mL of 10 % Folin-Ciocalteu reagent and 8.3 mL of distilled water were added to 0.2 mL of the 1 mg mL^{-1} extract solution, followed by vigorous shaking. Subsequently, 1 mL of 7.5 % sodium carbonate solution was introduced into the mixture. After thorough agitation, the samples were incubated in the dark for 30 minutes. The absorbance was measured using a UV/Vis spectrophotometer (GTA-96, Global Trade Technology, Jaboticabal, Brazil) with quartz cuvettes at a wavelength of 760 nm. A standard gallic acid calibration curve (y = 0.0013x + 0.0346; R^2 = 0.9933) was used, with concentrations ranging from 10 to 400 µg mL^{-1}. All measurements were performed in triplicate, and the results were expressed as gallic acid equivalents per gram of lyophilized extract (mg EAG g^{-1}).

Quantification of Flavonoid Content

The total flavonoid content was determined using the colorimetric method described by Peixoto Sobrinho et al.[54], with modifications adapted from Soares et al.[55]. A 0.5 mL aliquot of the extract solution (1 mg mL^{-1}) was mixed with 0.5 mL of 60 % acetic acid, 2 mL of 20 % pyridine, and 7 mL of distilled water. In the dark, 1 mL of 5 % aluminum chloride was then added to the mixture, followed by vigorous agitation. The reaction was allowed to proceed for 30 minutes. Absorbance was measured using a UV-Vis spectrophotometer at a wavelength of 420 nm. A standard calibration curve of rutin (y = 0.0012x + 0.0016; R^2 = 0.9941) was employed for quantification, with concentrations ranging from 10 to 400 µg mL^{-1}. The assays were conducted in triplicate, and the results were expressed as milligrams of rutin equivalents (RE) per gram of lyophilized extract (mg RE g^{-1}).

Evaluation of Antioxidant Potential

Three methods were employed to assess antioxidant activity: i) the inhibition of the stable free radical 2,2-diphenyl-1-picrylhydrazyl (DPPH•), as described by Brand-Williams, Cuvelier, and Berset[56], with adaptations by Peixoto-Sobrinho et al.[57]; ii) the scavenging of the 2,2'-azinobis-3-ethylbenzothiazoline-6-sulfonic acid radical (ABTS•+), following the method outlined by Rufino et al.[58]; and iii) the Ferric Reducing Antioxidant Power (FRAP), as described by Rufino et al.[59].

DPPH

The scavenging activity of DPPH• free radicals was evaluated by mixing 0.5 mL of different concentrations of extracts or standards (5-200 µg mL^{-1}) with 3 mL of a 40 µg mL^{-1} DPPH• methanolic solution, in triplicate. The blank was prepared by replacing the DPPH• solution with methanol in the reaction mixture. The reaction mixture and blank were shaken and protected from light for 30 minutes. After incubation, absorbance was measured at 517 nm using a spectrophotometer, calibrated with methanol. A DPPH• solution (40 µg mL^{-1}) was used as the negative control. Antioxidant activity (AA) was expressed as the percentage of inhibition and calculated using equation 2:

$$AA(\%) = (Ac - (Aa - Ab))/Ac \times 100 \qquad (2)$$

Where **AA (%)** represents the percentage of antioxidant activity, **Ac** is the absorbance of the negative control, **Aa** is the absorbance of the sample, **Ab** is the absorbance of the blank.

The IC_{50} value, representing the concentration of the sample required to reduce the initial DPPH• concentration by 50 %, expressed in µg mL^{-1}, was determined by plotting the different concentrations against the % AA using calibration curves.

ABTS

Antioxidant activity was also assessed based on the ability to scavenge the $ABTS^{\bullet+}$ radical cation. For this analysis, the $ABTS^{\bullet+}$ radical was generated by combining 5.0 mL of a 7 mM ABTS stock solution with 88 µL of a 140 mM potassium persulfate solution, followed by incubation in the dark at room temperature for 16 hours. The resulting solution was then diluted with 100% ethanol to achieve an absorbance of 0.700 ± 0.005

at 734 nm. In the dark, 3 mL of the ABTS•$^+$ radical solution was carefully mixed with 0.3 mL of test samples (50 μg mL^{-1}), homogenized on a tube shaker, and left at room temperature, shielded from light, for 6 minutes. Absorbance was measured at 734 nm using a spectrophotometer. A standard Trolox calibration curve (100–2000 μM) was constructed to determine the sample concentration equivalent to 1000 μM of Trolox. The results were expressed as μM of Trolox equivalent per gram of lyophilized extract.

FRAP

For the determination of total antioxidant capacity by ferric reducing power (FRAP), the FRAP reagent was prepared by combining 25 mL of 0.3 M acetate buffer, 2.5 mL of a 10 mM TPTZ (2,4,6-Tris(2-pyridyl)-s-triazine) solution, and 2.5 mL of a 20 mM ferric chloride aqueous solution. Following this, 270 μL of distilled water and 2.7 mL of the FRAP reagent were added to 90 μL of the test sample (50 μg mL^{-1}). The reaction mixture was incubated in a water bath at 37°C for 30 minutes, shielded from light. Absorbance was measured at 595 nm using a spectrophotometer. A standard curve was constructed using ferrous sulfate to determine the sample concentration equivalent to 1000 μM ferrous sulfate. Results were expressed as μM of ferrous sulfate equivalent per gram of lyophilized extract.
